# HOW APPROPRIATE IS THE GOLD STANDARD FOR DIAGNOSIS OF AIRWAY OBSTRUCTION?

**DOI:** 10.4103/0970-2113.45276

**Published:** 2008

**Authors:** Ashutosh N. Aggarwal

**Affiliations:** Associate Professor, Pulmonary Medicine Postgraduate Institute of Medical Education and Research, Chandigarh E-mail:ashutosh@indiachest.org

Demonstration of a reduced FEV_1_/VC ratio on spirometry remains the universally accepted criterion for diagnosis of airway obstruction in routine clinical practice. Despite this, there is no consensus as to how this “reduction” should be defined. It has been an age old practice to use a fixed ratio as the cut-off for this purpose. Most commonly, a FEV_1_/VC ratio less than 0.70 or 0.75 is used to interpret an obstructive defect on spirometry. Even though there is no statistical or epidemiological basis for choosing 0.70 (or for that matter any other similar ratio) as a cut-off for this purpose, this practice remains engrained in usage worldwide.

The problems and errors of using fixed percentages of reference values were pointed out more than four decades ago.^1^ Subsequently it was suggested that statistically derived lower limits of normal should be preferred over fixed percentages while interpreting spirometry data.^2^ We have earlier shown that use of fixed percentage based cut-offs introduce unacceptable misclassification rates in interpretation of spirometry results.^3^ In an effort to standardize interpretation of lung function tests, American Thoracic Society (ATS) proposed its guidelines in 1991, and recommended that airway obstruction should be defined by a FEV_1_/VC (or FEV_1_/FVC) ratio below a certain lower limit of normal (LLN).^4^ This LLN could either be the value below the fifth percentile or the lower 95 percent confidence limit of the values from a reference population. The more recent ATS/European Respiratory Society (ERS) guidelines published in 2005 have largely reiterated this stand.^5^ However, several old and recent international initiatives, mostly focusing on chronic obstructive pulmonary disease (COPD), still recommend use of fixed percentages for this purpose. The ATS had earlier defined airway obstruction as a FEV_1_/FVC ratio less than 0.75.^6^ British Thoracic Society recommends documentation of both reduced FEV_1_ (<80% predicted) and FEV_1_/VC ratio below 70% to diagnose airway obstruction.^7^ The global initiative for chronic obstructive lung disease (GOLD) guidelines, first published in 2001 and thereafter updated annually, define COPD by a post-bronchodilator FEV_1_/FVC ratio below 0.70.^8^ The recent ATS/ ERS position paper on the standards for the diagnosis and treatment of COPD also recommends using post-bronchodilator FEV_1_/FVC <70% as a criterion for diagnosing COPD.^9^ Among all these different approaches, the GOLD guidelines are fast becoming a benchmark in diagnosis and management of COPD.

The GOLD guidelines aim towards uniformity and simplicity in the area of COPD diagnosis and management. To achieve this end, and for greater use at primary and secondary levels of health care, developers of these guidelines have kept the diagnostic criteria for identifying airflow limitation very simple. In fact, they have gone back in history and have again proposed use of a fixed cut-off of 70% for FEV_1_/FVC for this purpose. Practically speaking, a person's FEV_1_/FVC ratio is an individual figure that depends, among other factors, on his/her race, ethnicity, body built and age. Because of this, it is almost impossible to accurately predict an individual's normal FEV_1_/FVC ratio. It is generally accepted that FEV_ 1_/FVC ratio decreases with increasing height and advancing age. Logically, therefore, it is not possible to choose any single value that can reliably discriminate healthy from diseased individuals. For epidemiological and clinical purposes, we generally rely on figures derived from apparently healthy population. Statistically derived values that take into account a person's age, gender and built, and provide a lower estimate of range of normalcy, are clearly a better option.^1^,^2^ Thus the real point of concern is whether this simplicity of the GOLD philosophy is achieved at the cost of clinical and scientific validity?

If we focus on the general population, it becomes clear that several healthy people have FEV_1_/FVC ratio below 70%. The proportion of such individuals increases with advancing age. In a population-based study in north Italy, spirometry was performed on 1727 adults aged 25 to 73 years. 40.4% of those studied had FEV_1_/FVC ratio less than 75%, and 18.3% had a ratio less than 70%.^10^ Abnormal results were more frequent in men, smokers, and those older than 45 years. In another study on nearly 4000 elderly subjects in Norway, 21.1% men and 17.4% women aged 60-69 years, and 38.1% men and 26.2% women aged more than 70 years, had FEV_1_/VC ratio less than 70%.^11^ Another small study on 71 asymptomatic Norwegian never-smokers aged more than 70 years showed FEV_1_/FVC value below 70% in 35% subjects overall, and 50% subjects aged above 80 years.^12^ It is pertinent to note that a large number of subjects suspected to have COPD are screened in the sixth decade or later. If the GOLD guidelines were to be followed, many of them may be falsely diagnosed as having airflow limitation based on a cut-off value of 70%.

**Fig 1:**
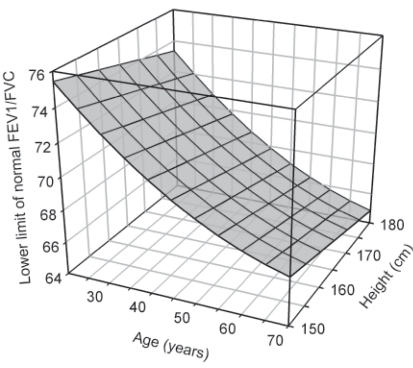
Lower limit of normal FEV_1_/FVC as a function of age and height in north Indian men. See references 13 and 14 for details on reference equations and mathematical calculations

Use of lower limits of normal derived from regression equations provide a kind of floating estimate of airflow limitation. Although this method is not a perfect solution, it is certainly much better in statistical terms in identifying a truly decreased FEV_1_/FVC value. As an example, regression equations based on gender, age and height have earlier been derived at our centre.^13^ Lower limits of normal are calculated by subtracting 1.645 times the standard error of estimate of the equation from the predicted value.^4^,^5^,^14^ Lower limit of normal FEV_1_ /FVC therefore varies with age and height, and we have earlier shown that this value is significantly different from 70% in a large proportion of patients undergoing spiromtery at our centre, more notably among women and elderly.^3^ Looking at estimates in men (Fig 1), it is immediately apparent that it is perfectly normal for a north Indian male aged more than 40 years to have a measured FEV_1_/FVC ratio below 70%, yet above the lower limit of normal for his age and height. As per standard guidelines for interpretation of spirometry, he would have normal lung function, but would be diagnosed as having COPD using the GOLD criterion.^5^,^8^ Clearly therefore, the GOLD strategy tends to overestimate airway obstruction in precisely the age band where it is most crucial to both diagnose as well as rule out COPD. By the same standards, these criteria would also underestimate airway obstruction in younger individuals in whom the lower limits of normal FEV_1_/FVC are considerably higher than 70% (Fig 1). In these individuals, a FEV_1_/FVC ratio above 70% could still be associated with true airway obstruction. And this point is not merely theoretical. Several investigators have indeed shown high misclassification rates using the GOLD guidelines. The largest of these efforts are based on the information from the Third National Health and Nutrition Examination Survey (NHANES III) database, and three separate analyses have been performed. In a study on 9838 subjects aged 30-80 years, abnormal FEV^1^ /FVC was defined as either a value below 70% or below the lower 95% confidence limit of predicted normal.^15^ Airway obstruction was diagnosed in 18.4% and 15.6% subjects respectively using the two criteria. The fixed percentage method overestimated airway obstruction by more than 1.5 times in adults aged 60 years or more. In another analysis of 13842 subjects aged 20 to 80 years from the NHANES III database, the number of individuals classified as mild and moderate COPD using GOLD strategy was increased by 58% and 37% respectively in the 50-80 years age category.^16^ In another study on 5906 smokers and 3497 nonsmokers from the same database, use of GOLD criterion resulted in 11.3% false positive results of airway obstruction.^17^ Other small studies also reflect similar results. In one study on 1503 hospitalized patients at Indianapolis, there was nearly 7% discordance between 70% criterion and reference equation derived lower limit of normal; this discordance increased with extremes of age on either side.^18^ In a similar study on 166 hospitalized patients in Philadelphia, 48 discordant results were obtained while using 70% and lower fifth percentile of predicted normal criteria; 43 (89.6%) of these were false positive results by the 70% criterion.^19^ In another study on 525 asymptomatic male smokers in Hong Kong, use of GOLD criterion as opposed to lower fifth percentile of predicted normal increased prevalence of airway obstruction from 17.8% to 45.4% in the 60-80 year age group.^20^ In another study on 749 adults from New Zealand, use of GOLD strategy increased age adjusted prevalence of COPD from 9.0% (derived using lower fifth percentile of predicted normal) to 14.2% in subjects aged 40 years or more.^21^

The GOLD criterion of using FEV_1_/FVC value below 70% as an indicator of airway obstruction cannot therefore be applied as a general ‘rule of thumb’ to the general population, as it will be associated with false negative results in young adults, and with false positive results in older adults. More importantly, prevalence of COPD may get artificially inflated in those aged more tan 40-50 years as the GOLD criterion fails to take into account that the FEV_1_/FVC ratio varies inversely with age and height. All evidence-based research, and expert recommendations from academic committees, recommend using statistically derived and ethnically appropriate “lower limit of normal” values for interpretation of lung function. Since none recommend the use of a fixed percentage criterion, it is time that we look again at our strategy in this area. Mathematical complexity, and lack of appropriate reference equations, have often been proposed as reasons by physicians and researchers who opt for the much simpler fixed percentage criterion. But these certainly are not such great problems that would justify the huge health costs associated with misdiagnosis of airway obstruction in a rather large proportion of our adult population.
